# Leveraging machine learning algorithm to predict minimum dietary diversity among children aged 6–23 months in Ethiopia

**DOI:** 10.1371/journal.pgph.0005995

**Published:** 2026-02-26

**Authors:** Naol Gonfa Serbessa, Siraj Muhidin Degefa, Beriso Alemu Hailu, Geleta Nenko Dube, Betelhem Bizuneh Asfawu, Asmamaw Ketemaw Tsehay, Eskedar Ayehu, Mulusew Andualem Asemahegn, Agmasie Damtew Wale, Eden Ketema Woldekidan, Tigist Tolessa Sedi, Asmamaw Deneke, Zehara Jemal Nuriye, Mohammedjud Hassen Ahmed, Habtamu Alganeh Guadie

**Affiliations:** 1 Department of Health Informatics, College of Health Science, Mattu University, Mattu, Ethiopia; 2 School of Public Health, College of Medicine and Health Sciences, Bahir Dar University, Bahir Dar, Ethiopia; 3 Department of Health Informatics, School of Public Health, Asrat Woldeyes Health Science campus, Debre Berhan University, Debre Berhan, Ethiopia; 4 Department of Health Informatics, College of Medicine and Health Science, Arbaminch University, Arbaminch, Ethiopia; 5 Department of Health Informatics, Arbaminch Health Sciences College, Arbaminch, Ethiopia; 6 Department of Health Informatics, School of Public Health, College of Medicine and Health Science, Wollo University, Dessie, Ethiopia; PLOS: Public Library of Science, UNITED STATES OF AMERICA

## Abstract

Lack of nutrient-rich food consumption is considered an important underlying factor affecting the healthy development of children, and can lead to developmental delays and various disorders. There is limited evidence on the predicators of dietary diversity. We aimed to train and test eight machine learning algorithms in the Ethiopian demographic and health survey (EDHS) from 2005–2019. We used secondary data from EDHS 2005, 2011, 2016 and 2019. A total of 8,996 weighted samples of children aged 6–23 months were included in the study. STATA 17 was used to extract variables from the EDHS dataset. Python 3.11 software was used for data cleaning, coding, and further analysis. The machine learning algorithms used in this study were logistic regression, random forest, K nearest neighbor (KNN), multilayer perceptron (MLP), support vector machine, naive Bayes, extreme gradient Boost (XGBoost), and AdaBoost. Furthermore, Shapley additive explanation’s (SHAPs) were used for model interpretability and to identify top predictors. The random forest classifier (accuracy = 82%, recall = 84.9%, precision = 78.5%, F1-score = 81.7%, area under the curve: AUC = 89%) was the best model for predicting minimum dietary diversity among children aged 6–23 month. Minimum Dietary Diversity is still a significant public health issue in Ethiopia, and there are important inequalities in regional and socioeconomic factors. The random forest model performed better for prediction and found place of delivery, sex of the household head, water source, place of residence, age of the child, number of children under five years of age, women’s years of age, and household size as the most important predictors. The result shows the importance of the use of machine learning in detecting the most-at-risk population and informing specific nutrition interventions.

## Background

“Minimum dietary diversity (MDD) is one of the main essential guidelines to prevent micronutrient deficiency among children from the eight-core infant and young child feeding (IYCF) recommendations to prevent child development faltering” [1]. According to the World Health Organization (WHO), minimum dietary diversity includes eight food groups, from which a child needs to consume at least five food groups to receive the optimal level of food required for their overall growth [2], including breast milk, grains, roots, and tubers; legumes and nuts; dairy products (infant formula, milk, yogurt, and cheese); flesh foods (meat, fish, poultry, and liver and organ meats); eggs; vitamin A-rich fruits and vegetables; and other fruits and vegetables [3,4].

Globally, inadequate diets and their implications continue to be a significant barrier to long-term socioeconomic growth and poverty reduction [5,6]. Poor nutrition takes the lives of 3.1 million children under the age of five each year worldwide [7]. A lack of nutrient-rich food consumption is considered an important underlying factor affecting the healthy development of children, which can lead to developmental delays and various disorders [8].Nutritional diversity is commonly acknowledged as a vital component however, only 29% of children aged 6–23 months have access to requirements for dietary diversity around the globe [9]. The period of 6–23 months is crucial for children’s growth and development, as it is known as the window of opportunity in the first 1000 days of life [10]. Nutritional supplements during the first 1000 days of a child’s life significantly improve birth and growth outcomes, preventing newborn stunting and laying the foundation for optimal development [11]. Malnourished children have a weakened immune system, increasing their vulnerability to illnesses such as malaria, respiratory infections, and diarrhea [12].

Research indicates that in several countries, fewer than 25% of children do not receive the necessary nourishment to develop properly. For example, in studies conducted in some Asian countries the prevalence of minimum dietary diversity (MDD) was 46.5% in Nepal [13]; in India, it ranged from 36.15–77% [14]; Indonesia, it was 46.7% [15]; and in Sri Lanka, it was 29% [16]. South Asian and sub-Saharan Africa accounted for the majority of the burden, meaning that the burden is not distributed equally. The largest burden of child malnutrition (57.7%) was found in Sub-Saharan Africa, particularly East Africa and West Africa, which indicated that children aged 6–23 months in Sub-Saharan Africa have a high prevalence of minimum dietary diversity intake [17]. According to research conducted in Ghana [18], Somalia [19], and Rwanda [20], the minimum amount of dietary diversity consumed was 35.3%, 15%, and 23%, respectively. Compared with other countries, Ethiopia is recognized for having a low MDD. According to reports, malnutrition among infants and young children in Ethiopia is a serious public health issue [21]. The recent 2016 EDHS showed that only 14% of Ethiopian children aged 6–23 months met the minimum requirements of dietary diversity, despite nearly half (45%) of them having an optimal frequency of meals [22]. In Ethiopia, the potential gap in minimum dietary diversity intake among children may result from low levels of food production and consumption, people’s living standards, adherence to national policies, sociocultural norms and attitudes, the availability of maternity and child health services, and people’s capacity to buy a variety of food items. According to the 2019 EDHS, 37% of children were stunted, 7% were wasted and 21% were underweight which represents a continuum of both acute and chronic malnutrition. Furthermore, only 7% of children are fed a minimum acceptable diet [23]. Community-based studies conducted in Ethiopia revealed that the minimum dietary diversity was 24.4% [24], 18.2% [25], and 21.8% [26]. The Sustainable Development Goal-2 aims to reduce malnutrition by 2030. The Ethiopian government responded by launching Growth and Transformation Plan II, the Second National Nutrition Program, and the Seqota Declaration. However, malnutrition in infants aged 6–23 months remains high [27].

Previous studies revealed that the age of a child [24,28], sex of a child [15], birth order [6,29–31], birth interval [24], breast feeding status [32,33], weight at birth [34], twin status [35], maternal age [15,36], maternal educational status [24,37–39], marital status [40,41], household head [42], number of children under-five [43], place of residence [44–46], media exposure [34,47], source of drinking water [11], ANC and PNC visit [17,28], place of delivery [36,48], household size [29,49] and household wealth status [50] were significant predictors of minimum dietary diversity.

Although few studies have been conducted in Ethiopia that primarily focused on measuring the levels of minimum dietary diversity via traditional statistical analysis techniques. Unlike traditional studies our research employed advanced supervised machine learning algorithms to uncover hidden predictors of MDD among children aged 6–23 months, providing a novel approach in the context of Ethiopian nutritional system Machine learning is increasingly used in public health research to overcome the limitations of traditional linear models and complex nonlinear data. Therefore, this research aimed to predict minimum dietary diversity and identify associated factors among children aged 6–23 months via a machine learning algorithm.

## Method and material

### Study design and period

A population-based cross-sectional study design was used in the EDHS. The study was conducted with the Ethiopian Demographic Health Survey (EDHS) dataset. The secondary data used in this research were taken from the EDHS 2005, 2011, 2016, and 2019. This study was conducted from December 2023 to July 2024 in GC.

### Study area

This study was conducted in Ethiopia. Ethiopia is located in the Horn of Africa and has a population of 120.3 million people, making it the second-most populous country in Africa. Ethiopia covers an area of approximately 1.1 million square kilometers and is bordered by Eritrea, Djibouti, Somalia, Sudan, and Kenya. Approximately 16% of Ethiopia’s population, or 13 million children under the age of five, live there. Nine regional states and two administrative cities constitute Ethiopia. Each region is further divided into zones, districts (Woreda), towns, and kebeles [5,6,15,27,51–53].

### Source and study population

#### Source population.

The source population of this study was all Ethiopian mothers who had children aged 6–23 months.

#### Study population.

All mothers who had children aged 6–23 months living in Ethiopia and who randomly chose enumeration areas during the survey year were considered.

### Inclusion and exclusion criteria

The study included all eligible mothers living in Ethiopia who had more than one child in the two years prior to the survey. Children aged 6–23 months with missing ages and those who died were excluded from the study. As a result, children who did not live with their mothers throughout the survey and those who provided incomplete information about MDD were excluded from the study.

### Data source, sample size, and sampling procedure

#### Data source.

The EDHS is a nationally representative survey that is carried out in Ethiopia every five years to supply accurate and current data on population and health-related issues in the nation. The data were obtained from the demographic health survey website (http://www.dhsprogram.com) through a formal online request that included an explanation of the research objective.

#### Sample size determination.

For this study, a total of 8,996 sample weights of children aged 6–23 months were extracted from four surveys (EDHS- 2005, 2011, 2016, and 2019). The children’s KR files were employed for this analysis. The survey included all nine regions and the two administrative cities of Ethiopia. Participants were selected on the basis of a stratified two-stage sampling technique from each enumeration area in all four survey years.

### Sampling method

A two-stage stratified sampling technique was employed by the DHS to choose a nationally representative sample that is proportionate to the population size of each regional state in the nation. For every round, the most recent housing and population census was used to prepare the sample frame. A total of 540 Enumeration areas (EAs) in the EDHS 2005,624 EAs in the 2011 EDHS, 645 EAs in the EDHS 2016, and 305 EAs in the 2019 EMDHS were randomly chosen in the first stage. In the second stage, a systematic sampling technique was used to select households proportionately from each EA.

### Study variables

#### Dependent variable.

The dependent variable was minimum dietary diversity which was categorized as 0 = adequate minimum dietary diversity and 1 = inadequate minimum dietary diversity. During the survey, their mothers were asked questions regarding the types of food the child ate throughout the day or night before the interview. The respondents are asked to specify whether their child ate anything from any of the eight food groups during the preceding day. The WHO recommended cutoff point was used to construct the minimum dietary diversity indicator from the dietary diversity score.

#### Independent variables.

**Fig 1:**
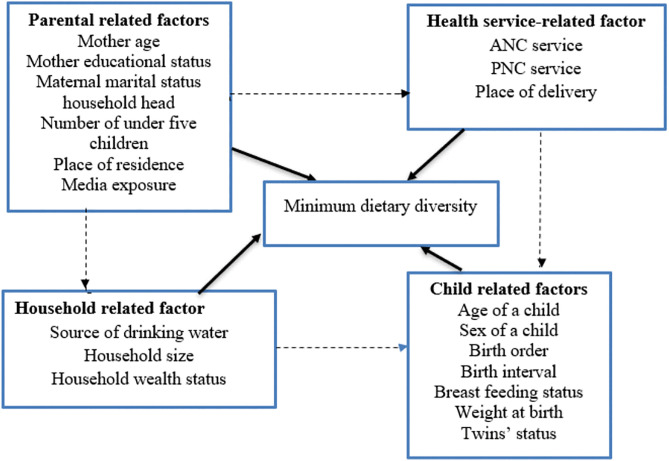
Conceptual framework for minimum dietary diversity among children aged 6-23 month.

Independent variables grouped as child factors include age, sex, birth order, birth interval breastfeeding status, twin status, birth weight, and sociodemographic factors of a mother, such as the mother’s educational status, the age of the mother, the head of the household, the marital status of the mother, the number of children under-five years of age, media exposure, the place of residence and health service factors, including ANC and PNC visits, the place of delivery and household factors such as the source of drinking water, household size and household wealth status. In order to clarify the rationale behind our choice of variable we incorporated those variables into conceptual framework Fig 1.

### Operational definition

**Minimum dietary diversity:-** Minimum dietary diversity is defined as the percentage of children aged 6–23 months who consumed at least five food groups out of the eight reference food groups in 24 hours [1]. These food groups are (1) breast milk; (2) grains, roots, and tubers; (3) legumes and nuts; (4) dairy products; (5) flesh foods (meats/fish/poultry); (6) eggs; (7) vitamin A-rich fruits and vegetables; and (8) other fruits and vegetables. According to WHO recommendations, a child’s score on the overall dietary diversity scale is between 0 and 8 [1].

**Adequate minimum dietary diversity:** Adequate minimum dietary diversity is defined as the proportion of children aged 6–23 months who consume >=5 food groups from the eight-food group

**In Adequate minimum dietary diversity:** Inadequate minimum dietary diversity is defined as the proportion of children aged 6–23 months who consume fewer than 5 food groups from the eight recommended food groups.

**Media exposure:** A composite variable obtained from EDHS by combining whether a respondent reads newspaper/magazine, listens to the radio, and watches television with a value of “0=No” if women were not exposed to at least one of the three media, and “1=yes” if a woman had access/exposure to at least one of the three media [51].

**Water sources:** According to the WHO [54], improved drinking water sources are likely to be safeguarded against outside contamination. These included piped water, public taps, standpipes, tube wells, boreholes, safe dug wells and springs, and rainfall. Because the quality of bottled water is unknown, households that use bottled water for drinking are classed as using an enhanced source only if the water they use for cooking and hand washing is from an improved source. Unimproved water sources include unprotected wells, unprotected springs, surface water (e.g., rivers, dams or lakes), vendor-provided water, and tanker truck-provided water.

### Data collection tool and procedure

The questionnaires included sociodemographic, socioeconomic, and nutritional data of children adapted to the Ethiopian context on the basis of the measure Demographic Health Survey. Recoding of children primarily included information on sociodemographic and other household characteristics of the children and mothers/guardians/primary caregivers, as well as characteristics related to nutrition, environment, and health services. The child questionnaire was designed to collect data from all women between the ages of 15 and 49 years on a variety of issues. DHS recodes raw data in different databases. In this study, child recoding (KR) was employed and appended together to focus on children between 6 and 23 months of age, after which variables were extracted. Children younger than age of 6 months and older than the age of 24 months were excluded, and children who lived elsewhere and died were excluded from the dataset.

### Data management and analysis

After the data are obtained from the DHS through request, Stata 17 is used to extract, append, keep, and join the variables. Once data were extracted from EDHS 2005, 2011, 2016 and 2019 to reduce the impact of sampling bias, the data were weighted using the EDHS dataset’s sample weight v005 to restore representativeness and account for the sample’s nonproportional distribution among strata and regions during the survey process. Python 3.11 software was used for data cleaning, coding, and further analysis to identify predictors of minimum dietary diversity by applying different predictive machine-learning algorithms. In this study we used Yufeng Guo’s seven steps of machine learning by implementing the sci-kit-learn and xgboost packages in Python and Jupiter Notebook. Furthermore, association rule mining was used for factor analysis.

### Regional variation analysis

We performed a regional variation analysis utilizing the administrative region variable (v024) from the DHS dataset in order to evaluate spatial variations in food diversity throughout Ethiopia. For each region, we determined the percentage of children with inadequate MDD with average dietary diversity score (DDS). To determine if regional differences were statistically significant, we employed two methods. To determine whether there were significant regional differences in the distribution of inadequate MDD, a Chi-square test of independence was first used. Second, the mean DDS scores were compared between regions using a one-way ANOVA. For both tests, a significance level of 0.05 was used. Furthermore, to evaluate the national trend in minimal dietary diversity (MDD) among children aged 6–23 months, we pooled data from four rounds of the Ethiopian Demographic and Health Survey (EDHS) conducted in 2005, 2011, 2016, and 2019. This combined dataset allowed us to reveal trends over time using a standard definition of dietary diversity based on the WHO’s eight food category categories.

### Machine learning approach to predict minimum dietary diversity

**Fig 2:**
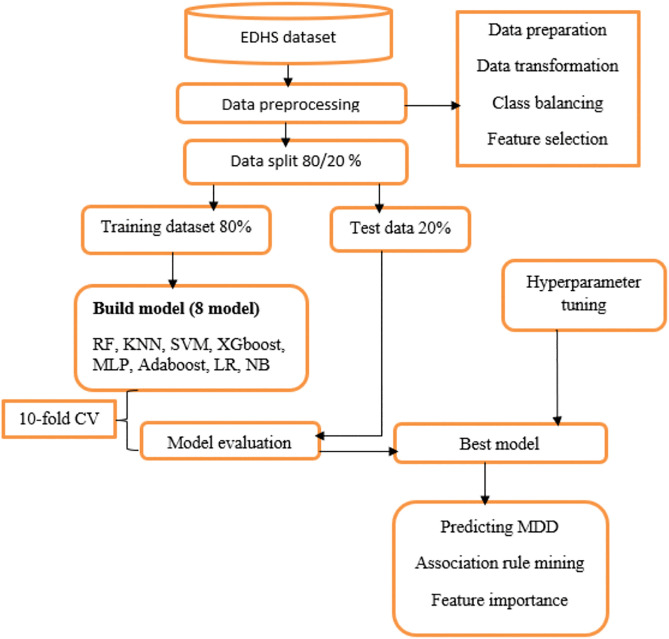
Flow chart of data preparation in machine learning.

This study used different types of supervised ML algorithms to identify predictors of minimum dietary diversity. In this study, we find predictors of children’s minimum dietary diversity (MDD) using machine learning models. It is crucial to remember that these models are not intended to prove causation; rather, they are intended to identify statistical correlations and prediction patterns in the data. While noting that additional research employing causal inference techniques would be required to validate causal effects, our study focuses on the predictive value of mother, child, household, and health service factors in connection to MDD. The seven steps of supervised machine learning algorithms include data collection, data preparation, model selection, model training, model evaluation, parameter tuning, and prediction [55] Fig 2.

### Data preparation

The dataset for this study is available on the measure demographic and health survey website obtained through a formal request. The dataset contains 8,996 weighted samples of 6–23 aged children. The target (outcome) variable of this study was minimum dietary diversity which is categorized as adequate MDD (“0”) or inadequate MDD (“1”). Machine learning algorithms require specific data formats, requiring preparation for useful insights. In this study, data were prepared from EDHS through the extraction and recoding of different variables from the dataset. Good data preparation creates clean and well-curated data, resulting in more practical, accurate model outcomes. The data preparation method is described as below.

### Data cleaning

Since the DHS dataset has missing values that can be encoded as NaNs, blanks, undefined, null, or any other placeholder, there are numerous techniques for substituting missing values including model-based handling of missing values, missing data imputation, and case deletion (row). In this study we evaluated the amount and distribution of missing data for each variable prior to developing the model. Birth weight (1,503 missing data), birth interval (1,722), antenatal care (ANC) visits (268), and postnatal care (PNC) visits (808), were among the important predictors with non-negligible levels of missingness. These variables, which are essential to mother and child health, were missing data, necessitating careful imputation to prevent selection bias and information loss. Missing data patterns in DHS surveys are generally considered to be missing at random (MAR), although specific variables such as birth weight and infant feeding practices can be missing not at random (MNAR), which may lead to biased results. We therefore performed sensitivity analyses. In this study, sensitivity analysis was conducted by comparing the performances of four techniques of imputing missing data such as multiple imputation by chained equations (MICE), K-nearest neighbors (KNN) imputation, Complete Case Analysis, and median imputation. The mean F-scores and standard deviations across folds of cross-validation, with logistic regression as the baseline model, were utilized for comparing the performances of all imputation techniques, which all performed equally well with a mean F1 Score of (0.903) for all models (see S1 Fig). Therefore, KNN imputation was selected for further analysis owing to its stability, efficiency, and ability to scale to large DHS data sets. KNN imputation is intended to identify the k nearest neighbors for an incomplete instance (missing data) among all complete instances (without missing values) in a given dataset. If the target feature (or attribute) is categorical, the most frequent neighboring value known as the majority rule, or the mean of the neighbors, known as the mean rule is used to fill in the missing datum. Previous studies [56,57] have successfully employed the KNN approach because of its simplicity, ease of understanding, and relatively high accuracy. KNN imputation was done with k = 5 nearest neighbors based on the Euclidean distance metric.

### Class balancing technique

To fix unbalanced categories of outcome variables the synthetic minority oversampling technique (SMOTE) is used to fix class imbalance. The outcome variable minimum dietary variety has a significantly unbalanced distribution in our dataset. In our data, 1,519 samples were classed as “adequate MDD,” while 7,137 were classified as “inadequate MDD”. This results in around 17.55% adequate and 82.45% inadequate cases, indicating a substantial class imbalance. This imbalance has the ability to significantly bias classification model performance in favor of the majority class, resulting in deceptively high accuracy with poor performance in the minority (adequate). To address this, we used the Synthetic Minority Oversampling Technique (SMOTE) during model training to artificially boost the representation of the minority class. This approach tries to increase model sensitivity and generalizability for the underrepresented “adequate” category.

### Feature engineering

The process of choosing, modifying, and converting unprocessed data into features that can be applied to supervised learning is known as feature engineering [58]. This process comprises creating features, transforming them, extracting features, analyzing exploratory data, and benchmarking. In this study, to screen out features on the basis of specific criteria, we employed a variance threshold feature selection method in conjunction with domain knowledge from nutrition science. The variance threshold is univariate selection technique that falls under the category of filter methods for feature selection which was used to eliminate features with near-zero variance, unlikely to provide any predictive information. If the variance of a feature is larger than the threshold value, the feature is kept; otherwise, it decreases However, we acknowledged that such strictly statistical filtering may unintentionally eliminate factors that are thematically important in the context of maternal and child nutrition. To address this, we augmented the statistical technique with expert-informed feature selection guided by reliable sources in public health and nutrition. We referred to the Demographic and Health Surveys (DHS), World Health Organization (WHO) standards, and Ethiopia’s National Nutrition Program (NNP) to preserve factors with known relevance to dietary variety outcomes. Among various feature engineering techniques, one hot encoding was used to encode categorical variables and create dummy variables.

### Dimensionality reduction

Dimensionality reduction is the process of reducing the number of features in a dataset while maintaining the highest level of information. This can be used to simplify a model, enhance the effectiveness of a learning algorithm, or facilitate data visualization [59]. In feature selection, the independent variables that are most important for predicting the target variable are chosen after employing statistics to evaluate the relationship between the independent variables and the outcome variable.

### Data split

Splitting datasets is regarded as an essential procedure for removing or minimizing bias in training data for machine learning models. The purpose of splitting data into different parts such as training and testing, is to avoid overfitting and model selection bias [60]. In this study, the 80/20 split method was employed, where 80% of the data were used for training and the remaining 20% were used for model testing. However, because large amount of data is not wasted when the number of samples is small, the tenfold cross-validation method was employed in this study for model training. Cross-validation is a technique for validating model efficiency by training it on a subset of input data and testing it on a previously unseen subset of the input data. It is also a method for determining how well a statistical model applies to a different dataset.

### Model selection

Model selection is an important step in developing efficient and accurate predictive models in the field of machine learning. Once the data were prepared and divided into training and testing datasets, a suitable model was selected for training. Since the outcome variable is categorical, the task is a classification task, and an appropriate classifier was selected to conduct the prediction. The classification algorithms used in this study were logistic regression, random forest, KNN, multilayer perceptron neural network, support vector machine, naive Bayes, extreme gradient Boost (XGBoost), and AdaBoost classifiers. These algorithms are selected on the basis of previous studies [56,60–64] since because of their simplicity and interpretability, they are suitable for baseline comparison and are efficient for handling binary classification in large datasets. If feature scaling is carried out, it can be effective for large datasets such as the DHS. A stratified 10-fold cross-validation was carried out, where the dataset was split evenly into ten non-overlapping subsets, and the original distribution of the class for the target variable was retained in each subset. In each subset, the developed models were trained on the other nine.

### Logistic regression (LR)

Logistic regression (LR) is a machine learning classification technique that employs logistic functions for binary dependent variables. In this study, the outcome variable (target variable) is the minimum dietary diversity categorized as adequate (0) or inadequate (1) MDD. The model is trained by adjusting the coefficients of the independent variables to minimize the error between the predicted and actual probabilities.

### Random forest (RF)

The random forest is a supervised ensemble learning method that uses a decision tree as its foundation. RF improves classification and regression performance by using multiple decision trees, bootstrap aggregation, and random resampling to create bootstraps and combine results for prediction. The random forest algorithm was used in this study because of its special ability to handle high dimensionality, complicated relationships, and imbalanced datasets. It offers feature-importance insights and is generally resistant to overfitting.

### KNN

K-NN is a member of the supervised machine learning category and is a reliable and flexible classification technique. Because it is nonparametric, it does not rely on any rigid presumptions regarding the underlying data. The algorithm’s decision boundary is contingent upon a few input points and their specific locations. The KNN rule presents a new pattern that is categorized into the class of the k closest neighbors that has the greatest number of members [65]. KNN was used in this study because it is simple to use, effectively handles a variety of data types, and yields interpretable results.

### Support vector machine (SVM)

SVM is a widely recognized machine learning model technique utilized for both regression and classification tasks. SVM is an effective technique that has been utilized to address real-world binary classification issues. SVMs outperform other supervised learning techniques [66]. In this study, SVMs were employed because they are effective at handling high-dimensional data, which makes them appropriate for tasks involving many features (independent variables).

### Extreme gradient Boost (XGBoost)

Extreme gradient boosting is another tree-based technique that makes use of a gradient boosting framework. Compared with gradient boost machine (GBM), this technique requires fewer resources to solve large-scale classification problems. Compared with earlier models, it uses a more regularized algorithm formalization to provide greater control against overfitting [67]. In this study, XGBoost was used because of its capacity to handle large datasets efficiently because of its parallel processing capabilities and optimized algorithms. This can lead to faster training times and better scalability than other models can achieve.

### AdaBoost classifiers

Adaptive boosting is a machine learning algorithm that can be used for a variety of classification and regression tasks. It is a supervised learning algorithm used to classify data by combining several weak or basic learners (such as decision trees) into a strong learner. AdaBoost weights training datasets on the basis of accuracy of previous classifications.

### Model training

Model training in machine learning refers to the process of teaching a machine learning model to make accurate predictions on the basis of input data. In this study, during training the model was exposed to a large dataset with known outcomes (adequate and inadequate MDD) and the model learned to identify patterns and relationships in the data. In this study, the model was trained with 80% of the dataset, and 20% of the test data were used by the selected model for evaluation purposes. Only the features selected via feature selection techniques were subjected to classification algorithms.

### Model evaluation

In this study, the algorithm performance the ML classifiers was evaluated by using a confusion matrix. A confusion matrix is a table used to evaluate the performance of the classification model. It shows the number of true positives, false negatives, true negatives, and false negatives. The row of the matrix represents the actual class whereas, the column represents the predicted class. The diagonal element of the matrix shows the correct prediction whereas, the off-diagonal element shows the incorrect prediction. The confusion matrix provides valuable insight such as the F-1 score, accuracy, precision, and recall. The evaluation metrics are calculated via the following equations:


$$\text{Accuracy}=\frac{\text{TP}+\text{TN}}{\left(\text{TP}+\text{FP}+\text{FP}+\text{FN}\right)}$$

$$\text{Precision}=\frac{\text{TP}}{\left(\text{TP}+\text{FP}\right)}$$

$$\text{Recall}=\frac{\text{TP}}{\left(\text{TP}+\text{FN}\right)}$$

$$\text{F1 score}=\frac{\left(\text{2}*\text{precison}*\text{recall}\right)}{\text{precision}+\text{recall}}$$

To evaluate the model’s fairness and generalizability across important sociodemographic factors, we performed a subgroup performance analysis by presenting the overall F1-score of the selected model. The F1-score was calculated separately for each subgroup using the predictions made for each member of that group. We were able to spot differences in model performance using this method that might not have been visible using just aggregate measurements.

### Hyperparameter tuning

Hyper parameters, such as the learning rate, the number of neurons in a neural network, or the kernel size in a support vector machine are employed for controlling the learning process of the model. The goal of hyperparameter tuning is to find the values that lead to the best performance on a given task. Optuna optimization is arguably the most basic hyperparameter tuning method. With this technique, a model is built for each possible combination of all of the hyperparameter values provided, each model evaluated, and the architecture that produces the best results is selected [68]. In this study, Optuna was used for hyperparameter optimization for the selected machine learning model.

### Making predictions

This is the last phase of the machine learning approach, where all of the previously mentioned tasks are implemented. Estimating the outcome variable from independent factors is the process of prediction. In this instance, the determination of minimum dietary diversity was predicated on significant variables that have been previously identified. Considering several predictor variables, the optimal classifier with a predetermined accuracy was used to identify a child’s minimum dietary diversity status and whether they met the necessary minimum dietary diversity according to the WHO and UNICEF criteria.

### Temporal analysis of predictors of MDD (2005–2019)

Using a temporal interaction analysis methodology, this study examined the changes over time in the factors leading to children’s Minimum Dietary Diversity (MDD) using data from four rounds of the Ethiopian Demographic and Health Surveys (EDHS): 2005, 2011, 2016, and 2019. To assess both cross-sectional relationships and temporal fluctuations in predictor influence, we employed a survey-weighted logistic regression model in Stata, adding interaction terms between each predictor and the survey year. We used Stata’s `margins` and `marginsplot` commands to illustrate temporal trends for each predictor and estimate the predicted probability of MDD across survey years. We were able to evaluate the changes in each predictor’s relationship to MDD over time due to these predictions. The significant interaction terms ({i.year##i.var`) revealed temporal heterogeneity, meaning that the predictor’s influence changed over several survey years.

### Association rule mining

Association rule mining is a data mining approach used to identify strong associations between different variables in the data [69]. An association rule is made up of two parts: the IF (antecedent) and the THEN (consequent). In this study, after the model was developed and its performance was evaluated, the independent predictors was used to predict minimum dietary diversity. Although significant factors are employed to predict minimum dietary diversity, the predictive model does not demonstrate which categorical variables are mutually linked with minimum dietary diversity. As a result, association rule mining analysis (If/then) was utilized to identify strong associations between independent attributes via a priori algorithm method.

### Model interpretability

In this study, after the model was trained, Shapley Additive Explanations (SHAP) was employed to interpret the model output and to understand the contribution of each feature to the prediction. We used global SHAP to analyze the selected model behavior and uncover key predictors of minimum dietary diversity. SHAP is model agnostic which provides a single framework for calculating the average contribution of each feature to the model’s predictions throughout the whole dataset, allowing us to rank features based on their relative relevance [70]. We obtained global SHAP values for all input variables and utilized summary graphs to visualize each feature’s average impact on model output. This method enabled us to identify characteristics that played a consistent and important role in the model’s decisions.

### Ethical clearance

Ethical clearance was obtained from the ethical review board of Bahir Dar University, College of Medicine and Health Sciences according to protocol number 984/2024. Permission for data access was requested from the measure demographic and health survey through an online platform by meeting all requirements needed to access data from http://www.dhsprogram.com. The DHS confirmed that the survey was approved by the central statistical agency (CSA) ethical review board, and all the adult participants provided verbal informed consent, which was provided by a field supervisor. The interviewers explained the survey’s purpose to each participant’s parents or guardians under the age of 18, and written informed consent was obtained from them prior to data collection. The measure DHS provides the dataset by deidentifying the personal information. There are no names of individuals or household addresses in the data files.

## Results

### Sociodemographic and housing conditions of the study participants

A total of 8996 weighted samples of children aged 6–23 months were included in the study. Among the total children included in this study, half (50.82%) were female. Approximately 36.09% of the children were aged between 12 and 17 months. According to this study, the majority (85.34%) of the participating mothers lived in rural areas. Most mothers (31.06%) were aged between 25 and 29 old. The majority, 94.45% and 63.04% of mothers, were married and had no formal education respectively. Approximately 77.57% of them mass media, had access to mass media. In terms of the household characteristics with respect to economic status, approximately 44.24% of households were poor and nearly half (54.15%) of households has unimproved access to water sources. (Table 1)

**Table 1:** Sociodemographic and housing conditions of study participants for minimum dietary diversity among children aged 6–23 months in Ethiopia (EDHS 2005–2019, N = 8996).

Variables	Categories	Weighted Frequency	Weighted percent
Women’s age	15-19	556	6.18
	20-24	2,024	22.51
	25-29	2,795	31.06
	30-34	1,786	19.85
	35-39	1,203	13.37
	40-44	485	5.40
	45-49	147	1.64
Place of residence	Urban	1,318	14.66
	Rural	7,678	85.34
Mother education stat	no education	5,671	63.04
	primary education	2,664	29.61
	secondary education	434	4.82
	higher education	227	2.52
Marital status	single	53	0.60
	Married	8,497	94.45
	widowed	101	1.12
	Divorced	345	3.83
No of under 5 children	1_child	3,207	35.65
	2_children	4,431	49.25
	3 and above	1,358	15.09
Media exposure	No	2,017	22.43
	Yes	6,979	77.57
Sex of hh head	Male	7,827	87.00
	Female	1,169	13.00
Child sex	male	4,424	49.18
	female	4,572	50.82
Child age	6-11 month	3,166	35.19
	12-17 month	3,246	36.09
	18-23 month	2,584	28.72
Birth order	first born	1,710	19.00
	second to fourth born	3,981	45.25
	five and above	3,305	36.74
Twins’ status	single birth	8,809	97.92
	1st of multiple	92	1.03
	2nd of multiple	95	1.05
Current breast feeding	No	888	9.87
	Yes	8,108	90.13
Birth interval	< 24_month	1,275	17.53
	>=24_month	6,000	82.47
Birth weight	Not weighted	6,719	89.61
	>2.5 kg	103	1.38
	<=2.5 kg	676	9.01
Household size	2-6	5,757	63.99
	7-10	3,009	33.44
	More than 10	230	2.57
Household wealth status	Poor	3,980	44.24
	Middle	1,878	20.88
	Rich	3,138	34.88
Water source	Unimproved	4,871	54.15
	Improved	4,125	45.85

### Health service-related characteristics

With respect to health service characteristics, the majority (45.97%) of women had no ANC visits, and most deliveries (72.33%) took place at home. With respect to PNC visits, the majority (93.15%) of women who had children aged 6–23 months did not receive PNC check-ups within two months. (Table 2)

**Table 2:** Health service-related characteristics of minimum dietary diversity among children aged 6–23 months in Ethiopia (EDHS 2005–2019, N = 8996).

Variables	Categories	Weighted Frequency	Weighted Precent
ANC visit	No visit	4,014	45.97
	<4 visit	2,315	26.52
	>=4 visit	2,401	27.51
Place of delivery	Health facility	2,489	27.67
	Home	6,507	72.33
PNC visit	No	7,773	93.15
	Yes	571	6.85

### Dietary diversity and consumption patterns of food groups among children

**Fig 3:**
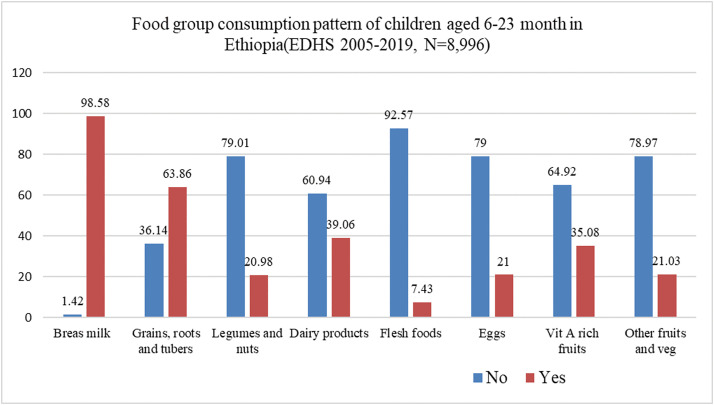
Dietary diversity and consumption patterns of food groups among children aged 6–23 months in Ethiopia (EDHS 2005–2019).

In this study, the consumption of food groups by children was based on the WHO eight-core infant and young child feeding (IYCF) recommendations. The majority (98.58%) of the children consumed breast milk, followed by grains, roots, tubers (63.86%), dairy products (39.06%), vitamin A– rich foods (35.08%), other fruits and vegetables (21.03%), eggs (21%), legumes and nuts (20.98%) and the lowest percentage of the children consumed flesh foods (7.43%) Fig 3.

### Trend of minimum dietary diversity

**Fig 4:**
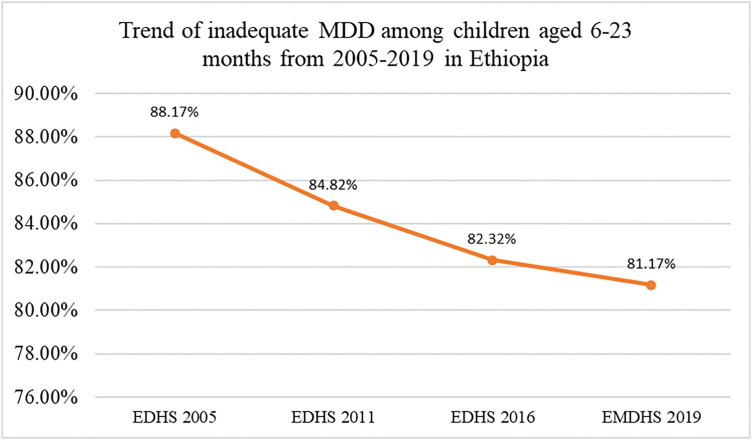
Trend of inadequate minimum dietary diversity among children aged 6–23 months in Ethiopia (EDHS 2005–2019), based on WHO eight food group categories.

In Ethiopia there was gradual improvement in children minimum dietary diversity between 2005–2019. The DHS data showed that the proportion of children aged 6–23 months with inadequate minimum dietary diversity declined from 88.17% in 2005 to 81.17% in 2019. This showed that there is consistent downward trend over the four EDHS survey periods (2005,2011,2016 and 2019). This suggested that, there is modest progress in improving child feeding practice throughout 15-year period Fig 4.

### Regional Variation of Minimum dietary diversity analysis

The percentage of children with inadequate minimum dietary diversity varied extensively by region. The regional differences in the prevalence of inadequate MDD were profound, with Oromia region having a prevalence of 26.2%, SNNP having a prevalence of 14.53%, and Somali having a prevalence of 10.68%. The mean of inadequate MDD scores showed differences between different regions. The Oromia region together with Addis Ababa reported the highest average scores at 3.3 and 3.4 while Gambella and Somali and Harari regions showed the lowest mean scores at 2.6 and 2.7 respectively. The Chi-square test showed that regional differences in inadequate MDD proportions were statistically significant (χ² (10) = 465.13, p < 0.001). The one-way ANOVA test detected significant regional differences in inadequate MDD mean scores (F (10, 8996) = 26.55, p < 0.001). This result showed that Somali and Harari and Gambella regions face severe dietary diversity challenge. (Table 3)

**Table 3:** Summary of regional variation analysis for minimum dietary diversity among children aged 6–23 months in Ethiopia (EDHS 2005–2019, N = 8996).

Region	Weighted Sample (2005–2019)	Percent of sample	weighted MDD status		Mean of Inadequate MDD Score across region
			Adequate MDD (%)	Inadequate MDD (%)	
Tigray	896	9.95%	252(15.56%)	644(8.73%)	2.9
Afar	749	8.32%	70(4%)	679(9.20)	2.8
Amhara	1,269	14.41%	395(24.39%)	874(11.85%)	3.1
Oromia	2,427	26.97%	494(30.51%)	1,933(26.2%)	3.3
Somali	821	9.13%	33(2.03%)	788(10.68%)	2.7
Benishangul	720	8%	158(9.75%)	562(7.61%)	3.0
SNNP	1,183	13.14%	111(6.86%)	1,072(14.53%)	3.2
Gambella	268	2.97%	31(1.91%)	237(3.21%)	2.6
Harari	257	2.85%	13(0.8%)	244(3.3%)	2.7
Addis Ababa	174	1.92%	41(2.53%)	133(1.8%)	3.4
Dire Dawa	232	2.56%	21(1.3%)	211(2.86%)	3.3
Total	8,996	100	1,619	7,377	

Chi-square test for proportion difference:*X*^*2*^(10) = 465.1345 p < 0.001

One-way ANOVA for mean MDDs: F(10, 8996) = 26.55, p< 0.001.

### Machine learning analysis for MDD

#### Feature selection.

**Fig 5:**
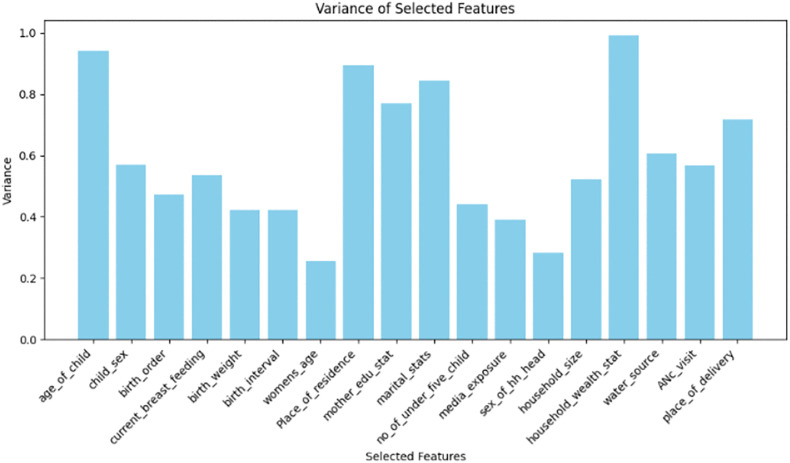
Variance threshold feature selection method for minimum dietary diversity among children aged 6–23 months in Ethiopia (EDHS 2005–2019, N = 8996).

In this study, the variance threshold feature selection method was used in order to select a subset of features from the overall feature space that allows a classifier to attain optimal performance. Variance threshold removes features whose variance does not meet some threshold. In this study, features with low variance (>0.01 threshold) such as PNC visit and twin status, were removed, and the selected features are shown in Fig 5.

#### Balancing data and model performance comparisons.

**Fig 6:**
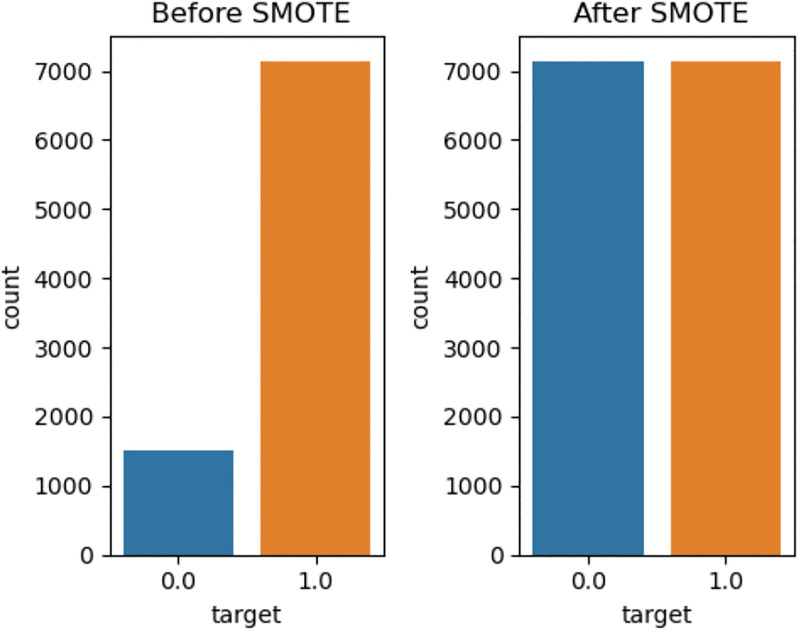
SMOTE balance of minimum dietary diversity among children aged 6–23 months (EDHS 2005–2016).

Balancing a dataset simplifies model training by preventing the model from being biased toward one class. In this study, the SMOTE balancing technique was used. The SMOTE oversampling strategy generated 5618 additional synthetic observations from minority groups with adequate MDDs to balance the unequal distribution of the target variable. To facilitate the creation of reliable prediction models and provide symmetric distributions for both groups, the total adequate minimum dietary diversity distribution was modified from 1519 adequate to 7,137 inadequate MDDs resulting in 7,137 in each class Fig 6.

For the unbalanced dataset, both logistic regression (accuracy of 83%, AUC 50%) and SVM (accuracy of 83%, AUC 50%) perform better than the other models do. However, the result was misleading because the model prefers the majority class simply because it has more data. After the SMOTE technique was applied to the imbalanced dataset, random forest was the best model, with an accuracy of 81%, an area under the ROC curve of 89% and F1- score of 82.3%. (Table 4)

**Table 4:** Comparisons of models that use accuracy and receiver operating curves for minimum dietary diversity among children aged 6–23 months in Ethiopia (EDHS 2005–2019, N = 8996).

NO	Models	Data status	Accuracy	F-1 score	AUC
1	Logistic regression	Unbalanced	0.83	0.90	0.50
		Balanced	0.67	0.65	0.71
2	Random forest	Unbalanced	0.82	0.89	0.60
		Balanced	0.81*	0.823*	0.89*
3	XGBOOST	Unbalanced	0.82	0.90	0.53
		Balanced	0.74	0.73	0.82
4	AdaBoost	Unbalanced	0.78	0.80	0.50
		Balanced	0.63	0.64	0.69
5	KNN	Unbalanced	0.81	0.89	0.53
		Balanced	0.74	0.71	0.83
6	SVM	Unbalanced	0.83	0.91	0.50
		Balanced	0.70	0.70	0.78
7	Naïve bayes	Unbalanced	0.20	0.09	0.51
		Balanced	0.63	0.70	0.64
8	MLP	Unbalanced	0.74	0.85	0.53
		Balanced	0.73	0.72	0.82

### Hyperparameter tuning

This study used Optuna for hyperparameter optimization in machine learning models. Optuna uses random and Bayesian samplers to determine optimal hyperparameter values. After the best model, which is a random forest classifier, is selected, the model undergoes optimization on n estimators (the number of decision trees in the forest, drawn from a sample between 100 and 500) which was 250 (95% CI: 243–267), max _ features (the number of features, selected from the category options [‘sqrt’, ‘log2’, None], in which each tree takes into account when a node is divided.) which was 0.15 (95% CI: 0.13-0.18), min samples split (The minimum number of samples needed to split an internal node) which was 4 (95% CI: 3–5), min samples leaf (the minimum number of samples needed to be at a leaf node, sampled from the range) which was 1(95% CI: 1–2), and max samples (the number of samples to draw from independent variables to train each tree) which was 0.99 (95% CI: 0.96-1.00). The optimized hyperparameters are stratified 10-fold cross-validated to maximize random forest performance. Finally, using balanced training data and fine-tuned hyperparameters, a random forest model was created via 10-fold cross-validation, which yielded an accuracy of 82% and an area under the curve of 89%. (Table 5)

**Table 5:** Default and optimal tuned hyperparameter of random forest for minimum dietary diversity among children aged 6–23 months in Ethiopia (EDHS 2005–2019, N = 8996).

Hyperparameter	Default	Optimal (mean ± 95% CI)
Number of trees (n_ estimator)	100	250 (243-267)
Number of features for best split (max_ features)	Sqrt (n_ features)	0.15 (0.13-0.18)
Min samples required to split internal node (mean_ sample_ split)	2	4 (3–5)
Minimum number of samples to be a leaf node (mean_ sample_ leaf)	1	1 (1–2)
Max sample to draw (max_ features)	None	0.99 (0.96-1.00)

### Feature importance visualization

**Fig 7:**
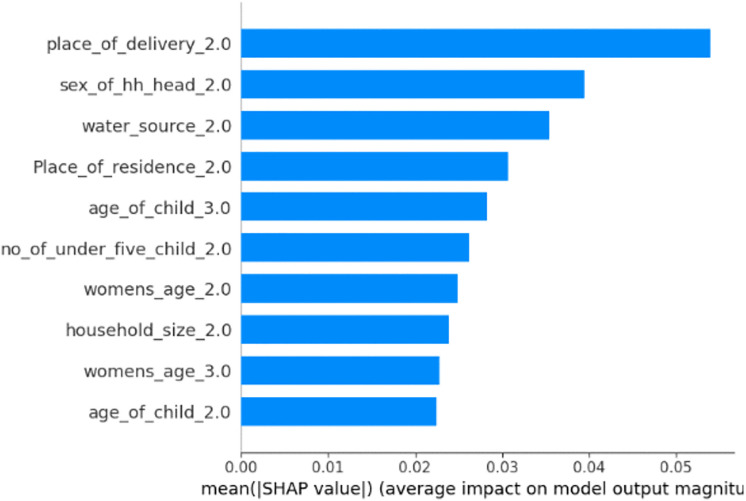
SHAP global importance plot of the optimized random forest model for minimum dietary diversity among children aged 6–23 months in Ethiopia (EDHS 2005–2019, N = 8996).

In this study, SHAP global feature importance was used to select the top predictors of minimum dietary diversity among children. The absolute summary plot from the random forest model is displayed. To create a bar chart that represents the contribution of each variable to the model’s prediction, the average absolute value of the SHAP values for each variable was determined. In this manner, the relative contribution of each variable to the prediction of the minimum dietary diversity among children aged 6–23 months was observed. This result suggests that, from the average SHAP value, the most important or influential variables in the model were Place_of_delivery_2 (children born at home), sex_of_hh_head_2 (female), water_source_2 (improved source), place of residence_2 (rural), age_of_child_3 (18–23 months), number of_under_five_children2 (2_children), womens_age_2 (20–24 years old), house_hold_size_2 (7–10 members), womens_age_3 (25–29 years old), and age_of_child_2 (12–17 months) Fig 7

### Model interpretation

The global importance plot does not show which feature positively or negatively affects the prediction. For this purpose, a summary plot using beeswarm was employed to elucidate the significance and relationship between each of the top ten features and the outcome variable. In the beeswarm plot the most significant features are at the top of the SHAP plot, which is created by sorting the ten highest–ranking features according to their mean absolute SHAP values in descending order. Each dot corresponds to one child in the study. The beeswarm graph demonstrates how the ML model identifies predictors of the minimum dietary diversity as it’s impacted by the various feature expressions of each child. Both positive and negative SHAP values show a shift in the expected model prediction in the direction of adequate minimum dietary diversity and inadequate minimum dietary diversity consumption, respectively.

**Fig 8:**
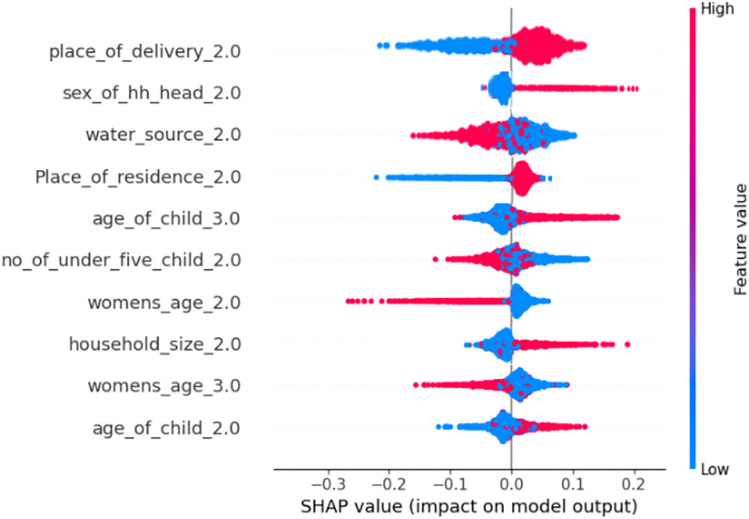
Beeswarm plot of the mean absolute SHAP value from the optimized random forest model for minimum dietary diversity among children aged 6–23 months in Ethiopia (EDHS 2005–2019, N = 8996).

The red and blue colors correspond to the variable’s higher and lower values for each predictor. Points to the right of the vertical line (0 SHAP value) indicate a greater chance of inadequate minimum dietary diversity, whereas points to the left indicate a lower chance of inadequate minimum dietary diversity. With respect to female household heads (sex_of_hh_head_2), having children born at home (place of delivery_2), having a child between the age of 18–23 (age of child_3), living with 7–10 family members (household_size_2) increase the likelihood of inadequate minimum dietary diversity intake. Having an improved water source (water_source_2), and having 2 children (no_of_under_five_child_2), decreases the likelihood of inadequate minimum dietary diversity Fig 8.

### Waterfall visualization

**Fig 9:**
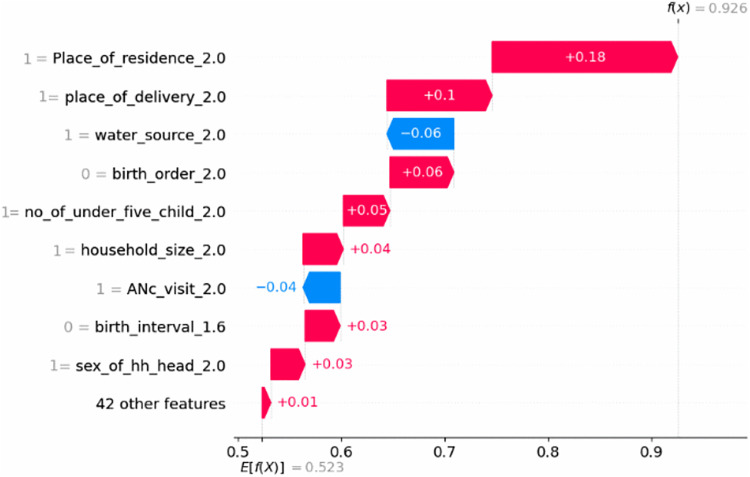
Waterfall plot visualization of the prediction of first observation for minimum dietary diversity among children aged 6–23 months in Ethiopia (EDHS 2005–2019, N = 8996).

This study used waterfall visualizations to explain model predictions for the first observation. The bottom of a waterfall plot begins with the expected value of the model output, and each row displays how the positive (red) or negative (blue) contribution of each feature shifts the value from the expected model output over the background dataset to the model output for this prediction. In this study, by the combining of the positive contributions (in red) and the negative contributions (in blue), if the model output is greater than this number (E [f(X)] = 0.532), it indicates a positive class (inadequate MDD), and if the score is less than this value, it indicates a negative class (sufficient MDD). As a result, for the first observation, the predicted value output is moved to the final model output (f(x) = 0.926), which is classed as a positive class (inadequate MDD), on the basis of lives in rural area (1 = place of residence_2), children who were born at home (1 = place of delivery_2), not having a second to fourth child born (0 = birth_order_2), having two children(1 = no_of_under_five_children), having 7–10 family members(1 = household size_2), having a preceding birth interval <24 months (0 = birth_interval_1.6) and being female household head (1 = sex of hh head) increases the probability of inadequate MDD intake while having an improved water source (1 = water_source_2) and ANC visit greater than four (1 = ANC_visit_2) decreases the probability of inadequate MDD Fig 9.

### Temporal analysis of predicted probability of MDD

The expected predicted probability of inadequate minimum dietary diversity (MDD) among children aged 6–23 months for each of the key variables over the course of four EDHS survey years are shown in (Table 6)

**Table 6:** Predicted probability of inadequate minimum dietary diversity by predictors (2005-2019 EDHS) among children aged 6-23 months.

Variable	2005 (95% CI)	2011 (95% CI)	2016 (95% CI)	2019 (95% CI)
sex of Household Head (Female)	0.41 (0.38–0.44)	0.43 (0.40–0.46)	0.46 (0.43–0.49)	0.50 (0.47–0.53)
Place of delivery (Home Delivery)	0.45 (0.42–0.48)	0.47 (0.44–0.50)	0.50 (0.47–0.53)	0.52 (0.49–0.55)
Age of a child (Child Aged 18–23 Months)	0.39 (0.36–0.42)	0.41 (0.38–0.44)	0.44 (0.41–0.47)	0.46 (0.43–0.49)
Household Size (7–10 member)	0.42 (0.39–0.45)	0.44 (0.41–0.47)	0.47 (0.44–0.50)	0.49 (0.46–0.52)
Water Source (Improved)	0.35 (0.32–0.38)	0.33 (0.30–0.36)	0.30 (0.27–0.33)	0.28 (0.25–0.31)
Number of Under-Five Children (Two children)	0.36 (0.33–0.39)	0.34 (0.31–0.37)	0.31 (0.28–0.34)	0.29 (0.26–0.32)

**Fig 10:**
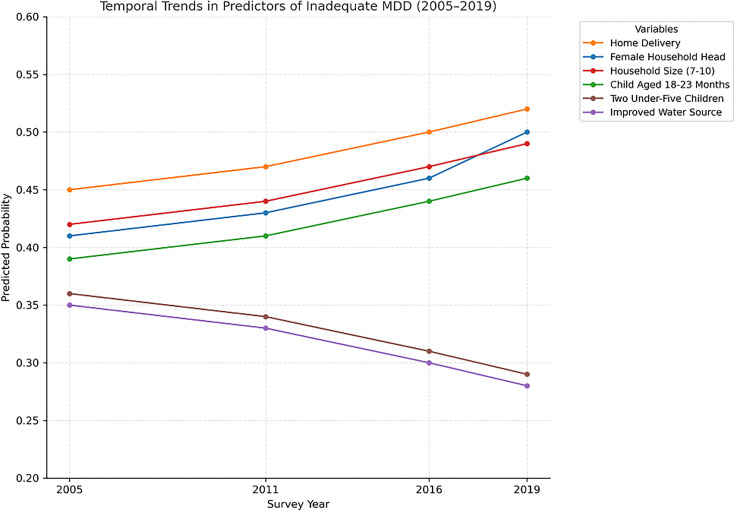
Temporal analysis of predictors minimum dietary diversity across survey years among children aged 6–23 months in Ethiopia (EDHS 2005–2019, N = 8996).

The influence of a number of predictors changed significantly over time, according to the temporal analysis. Children from families with female heads, children born at home, children between the ages of 18 and 23 months, and children from households with seven to ten members, for instance, were more likely to have inadequate MDD in all four surveys. Having two children under five and better water supplies, on the other hand, were consistently linked to a lower likelihood of inadequate MDD. For these important variables, the projected odds of inadequate MDD changed with time, as shown in the figure below Fig 10.

### Regional heterogeneity in predictors of MDD

In an attempt to address issues relating to contextual heterogeneity, region-stratified model explanation was performed utilizing SHAP values extracted from the final Random Forest model. It was observed that there is considerable variation in regions for individual predictor variables that impact inadequate minimum dietary diversity (See S2 Fig). It is seen that, while ‘Place of Delivery’ is generally a highly important feature, its significance is greater in regions that are urbanized as well as agrarian, for instance Addis Ababa (SHAP = 0.52) and Oromia (SHAP = 0.50). On the contrary, ‘Water Source’ turns out to be a highly important factor in regions that are pastoralist, for example, Afar and Somali, compared to overall national averages. Additionally, significance levels for ‘Sex of Household Head’ also vary considerably, indicating that ‘power dynamics’ for women in regions exert considerable weight compared to national averages, particularly for SNNPR and Addis Ababa.

### Predicting minimum dietary diversity

**Fig 11:**
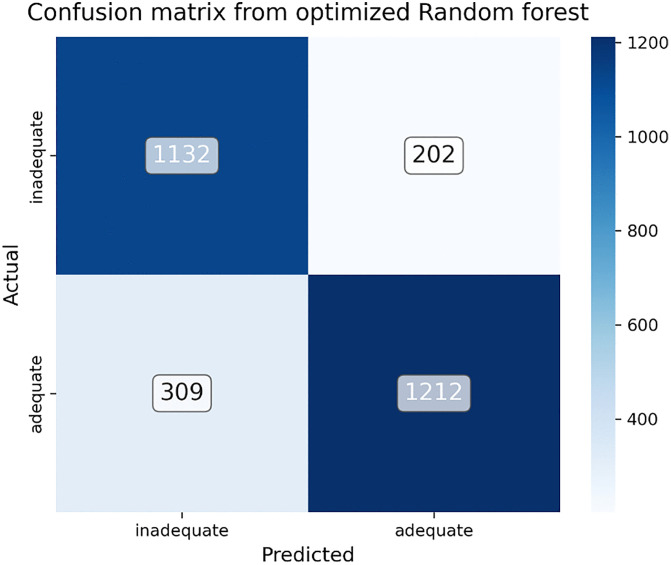
Confusion matrix from the optimized random forest on test data for minimum dietary diversity among children aged 6–23 months in Ethiopia (EDHS 2005–2019, N = 8996).


After training and optimizing the random forest model, 2855 test samples which were previously unseen data were predicted. Out of the 1,441 cases of inadequate minimum dietary diversity, the model correctly predicted that 1103 of the children met the inadequate minimum dietary diversity (true positive). However, 338 of them are incorrectly classified as adequate when they are inadequate (false positive). Out of 1414 individuals with adequate minimum dietary diversity, the model correctly predicts that 1,212 children meet adequate minimum dietary diversity (true negative), but 202 of them are incorrectly classified as having inadequate minimum dietary diversity (false negative). Overall, the model predicts minimum dietary diversity with an accuracy of 82.1%, precision of 78.5%, recall of 84.9% and F1- score of 81.7% Fig 11

$$\text{Accuracy}=\frac{\text{TP}+\text{TN}}{\text{TP}+\text{FP}+\text{FN}+\text{TN}}=\;\frac{\text{1132}+\text{1212}}{\text{1132}+\text{309}+\text{202}+\text{1212}}=\text{ 82}\%$$

$$\text{Precision}=\frac{\text{TP}}{\text{TP}+\text{FP}}=\;\frac{\text{1132}}{\text{1132}+\text{309}}=\text{ 78}.\text{5}\%$$

$$\text{Recall }=\;\frac{\text{TP}}{\text{TP}+\text{FN}}=\;\frac{\text{1132}}{\text{1132}+\text{202}}=\text{ 84}.\text{9}\%$$

$$\text{F1}=\;\frac{\text{2XprecisionXrecall}}{\text{precision}+\text{recall}}=\;\frac{\text{2X0}.\text{7855X0}.\text{849}}{\text{0}.\text{785}+\text{0}.\text{849}}=\text{ 81}.\text{7}\%$$

**Fig 12:**
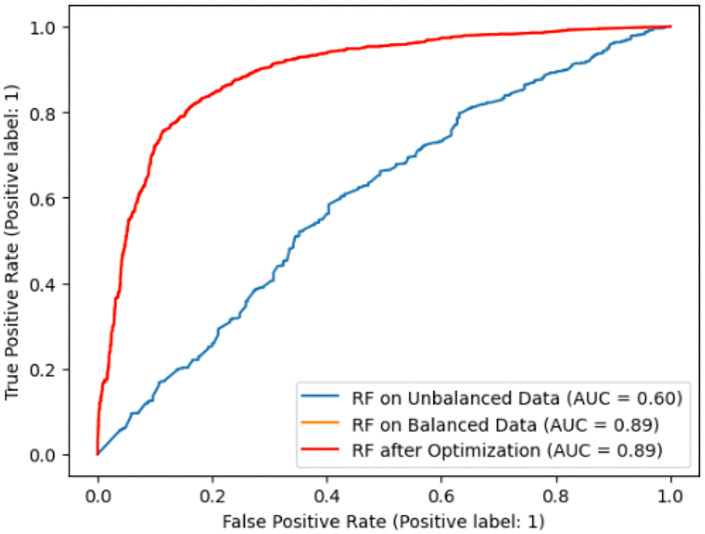
Comparison of random forest model predictions on test data for minimum dietary diversity among children aged 6–23 months in Ethiopia (EDHS 2005–2019, N = 8996).

Performance measurement is an important task in machine learning. Therefore, in regard to classification problems (adequate and inadequate MDD), the AUC - ROC curve is the best way to determine how well a model can distinguish between classes. The random forest algorithm created an area under the curve of 89% for both balanced data and hyperparameter tuning Fig 12

### Subgroup model performance

**Fig 13:**
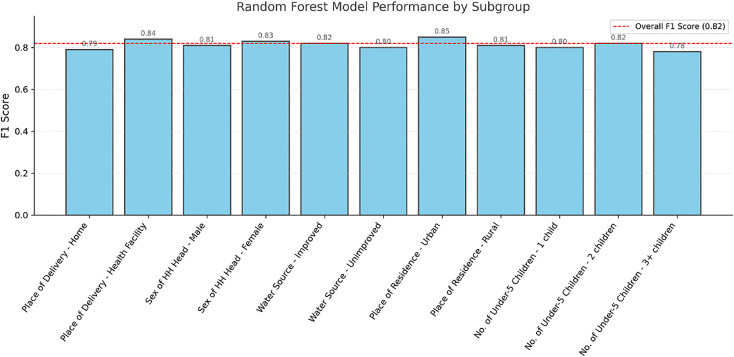
Subgroup performance evaluation of random forest model for minimum dietary diversity among children aged 6–23 months in Ethiopia (EDHS 2005–2019, N = 8996).

When the Random Forest model was evaluated against important household and demographic variables, it showed small but significant differences in performance. The model had the best F1-score for urban homes (0.85) and health facility deliveries (0.84). It had the worst F1-score for home deliveries (0.79) and residence with unimproved water (0.80). Performance was similar for families with different numbers of children under the age of five years (0.81–0.83) and between families led by a male (0.81) and female (0.83) household head. Overall model F1-score of 0.82 is indicative of overall stable performance Fig 13.

### Association rule mining

In this study, the a priori algorithm generates 67 rules related to category 1 (inadequate MDD), and the top five rules are listed below on the basis of their confidence and lift values. The most common antecedents (factors) strongly related to minimum dietary diversity were place of residence, water source, household size, place of delivery, age of a child, number of children under five, and women’s age.

Rule 1: **IF** ‘’household_size_2.0 = 7–10, no_of_underfive_children_2 = 2 children, child age_3 = 18–23” →**THEN** probability of target_1 = True (confidence = 83.5%, lift = 1.155)
Rule 2: **IF** ‘’place_of_delivery_2.0 = home, womens_age_2 = 20–24, household_size_2 = 7–10” → **THEN** probability of target_1 = True (confidence = 89.4%, lift = 1.399)
Rule 3: **IF** ‘’no_of_underfive_children_2 = 2 children, place of residence_2 = rural, child_age_2 = 12–17” → **THEN** probability of target_1 = True (confidence = 83%, lift = 1.352)
Rule 4: **IF** ‘’place of delivery_2 = home, women’s age_2 = 20–24, child age_2 = 12–17 month” →**THEN** probability of target_1 = True (confidence = 87.5%, lift = 1.32)
Rule 5: **IF** ‘’household size_2 = 7–10 members, no_of_underfive_children_2 = 2 children, place of residence_2 = rural” →**THEN** probability of target_1 = True (confidence = 90.5%, lift = 1.159)

## Discussion

The trends, variations, and machine learning predictive analysis of minimum dietary diversity (MDD) of children aged 6–23 months were explored in this study using four EDHS surveys conducted between 2005–2019. The findings present a linear but modest increase in MDD over the period of 15 years, which indicates gradual advancement towards improved infant and young child feeding. The persistent high prevalence of low MDD, however, is an indicator of the ongoing problem of ensuring diverse diets for young children. Regional variation was showed that, Somali, Harari, and Gambella having lowest MDD values, relative improvement in Oromia and Addis Ababa. Predictions via machine learning approaches are gaining popularity and importance in healthcare research because of their ability to manage massive datasets that are available. This study has proven the relevance of machine-learning methods to constructing prediction models on the basis of features discovered to be determinants in machine learning classifiers. In this study, eight machine learning algorithms were trained on both balanced and unbalanced datasets to determine the superiority of one of the best models. According to the model performance evaluation results in this study, data imbalance had an impact on the prediction performance on the hold-out or unseen dataset. In the present study, the RF model has greater predictive power than the other ML models, with an accuracy of 82%, F-1 score of 81.7%, precision of 78.5%, recall of 84.9% and area under the receiver operating curve of 89%. This performance was slightly lower than that reported in a similar study conducted in Bangladesh(accuracy 85%) [62]. This discrepancy might be due to differences in the dataset and features used to train the model. The other possible reason is that studies performed in Bangladesh utilized 70/30% of the training test split and did not perform feature selection. Finally, after hyperparameter tuning prediction was performed on the test data, the model predicted 1132 cases of truly inadequate MDD (TP) and 1212 cases of truly adequate MDD (TN) and misclassified 309 cases of inadequate MDD (FP) as adequate and 202 adequate MDD as inadequate MDD (FN).

The interpretability of the models is crucial for incorporating machine learning techniques into decision-making processes [71]. In this study, Shapley additive explanation (SHAP) was used to explain individual predictions by highlighting the essential features that caused that prediction. On the basis of RF model, the SHAP analysis revealed that having children who were born at home, being female household heads, having a child between the ages of 12–17 and 18–23, having children living with 7–10 family members, having improved water sources, living in rural areas, and having two under five children were the top predictors of minimum dietary diversity among children aged 6–23 months.

On the basis of SHAP model explanation, children born at home increase the likelihood of having inadequate minimum dietary diversity. This finding is in line with studies conducted in Dire Dawa [24] and Ghana [9], which reported that, in comparison with mothers who gave birth in a health facility, mothers who gave birth at home were more likely to fail to provide the recommended minimum dietary diversity for their children. This could be because mothers who gave birth in a health facility were more likely to attend antenatal clinics, where they were more likely to have good contact with health personnel who could provide them with information, knowledge and assistance on safe child feeding practices.

Being female a household head increases the likelihood of inadequate minimum dietary diversity of children. This evidence is supported by a study conducted in Wolaita Sodo town [42] and a cross-sectional study using EDHS 2016 [72], which reported that children who lived with a female household head were more likely to have inadequate minimum dietary diversity. This might be because many female heads find it difficult to sustain families with only one person’s income, especially if the female’s earnings are small (low socioeconomic status). The other possible reason is that the absence of a male earner and a lack of material resources generated a scenario of increased vulnerability for female heads of house hold which made them unable to provide the necessary and sufficient amount of food for their children.

This study also revealed that younger children aged 12–17 months were highly likely to have inadequate MDD. This result was supported by studies conducted in Nepal [13] and Indonesia [10], which revealed that, children aged 12–17 months have a greater likelihood of not having MDD. A possible explanation for this finding might be that many mothers believe that children under the age of one year do not require diver’s foods and should avoid animal-sourced food because the child’s gut does not digest such foods. They may also believe that most forms of food, including vegetables, fruits, and meat, are ingested more often at the age of 12 months. However, the findings of this study disagreed with those of a study conducted in three sub-Saharan African countries [44] that used demographic health survey data and reported that children aged 12–17 months were nearly twice as likely to have adequate MDD. This difference can be attributed to a variety of factors, including differences in agricultural productivity, economic situation, and cultural practices.

According to this study, children living in families ranging from 7–10 in size were highly likely to have inadequate MDD. This finding is in line with studies conducted in the Gedeo zone [73], Shamane [47], Rwanda [20] and Indonesia [49], which reported that children living in larger households may have a lower likelihood of meeting the necessary dietary diversity requirements. This might be due to the family’s capacity to meet its members’ nutritional needs, and as the number of family members increases, the intrahousehold food distribution is changes, and food may become more limited, which in turn limits access to different food categories.

This study revealed that children who live in rural areas are more likely to have inadequate minimum dietary diversity. This finding is supported by studies conducted by Bench Maji [50], Uganda [3] and Pakistan [74], which reported that children in rural areas are more likely to have inadequate minimum dietary diversity than are those in cities. This may be, because mothers who live in rural areas may be unaware of proper infant and young child feeding practices. Because rural mothers do not have diverse exposure to information, inadequate access to health services and counseling on child nutrition increases the need for diverse dietary feeding practices.

The waterfall plot indicated that women who had at least four ANC visits were less likely to have inadequate minimum dietary diversity. This finding is supported by studies conducted in Debrelibanos [75] and northwestern Ethiopia [17]. Mothers who attended ANC (antenatal care) visits during pregnancy were more likely to offer adequate nutritional diversity for their children. This could be due because mothers who had ANC visits may have obtained various health information related to child nutrition from their health care providers.

Another top predictor of minimum dietary diversity was having an improved water source. Those family members who have improved water sources are less likely to have inadequate minimum dietary diversity. This finding is supported by a study conducted in Ghana [76], which revealed that the availability of improved water source correlates with increased dietary diversity and better nutritional status in children. This is because improved water availability is frequently associated with improved hygiene and sanitation practices [4,14]. Furthermore, access to improved water sources at home saves time and resources that would otherwise be spent getting water from distant sources that may help to prepare a variety of foods from different groups for children. This can lower the likelihood of waterborne diseases and infections, resulting in improved overall health and nutritional status in children, even if their dietary diversity is limited.

### Strengths and limitations

The study’s strength is its analysis of a nationally representative sample of children aged 6–23 months over four years of EDHS data (2005–2019) with a high response rate. It employed the latest minimum dietary diversity indicator reflecting WHO recommendations to assess IYCF feeding practices effectively. Additionally, the use of SHAP enhances interpretability and reliability of model predictions by clarifying feature contributions and ranking their importance, thus identifying key factors influencing outcomes.

This study acknowledges limitations due to the use of secondary, cross-sectional DHS data. First, biases may arise from the mother’s recall of feeding practices. Second, the cross-sectional design makes it impossible to draw conclusions about causality, and the correlations found shouldn’t be seen as causal or temporal interactions. Third, the omission of key variables such as nutritional counselling, paternal characteristics, and childhood morbidity from the 2019 EMDHS dataset may restrict model performance and influence the interpretation of feature importance. Fourth, dietary taboos, gender norms, and seasonal variation in food availability are some of the culturally and contextually relevant predictors of child feeding practices, not captured in the DHS dataset. since their absence may further restrict the interpretability of the model. Lastly, coefficients and odd ratios are not presented directly from the machine learning prediction outcome despite the use of SHAP values. With respect to traditional statistical models, the strength of relationships between variables can hardly be described in the machine learning prediction outcome. Future studies, involving longitudinal, cultural, and environmental variables, might reveal more patterns concerning the effects of children’s nutrition.

## Conclusion

Minimum dietary diversity (MDD) is a major public health concern in Ethiopia. The finding shows that there are regional disparities in inadequate minimum dietary diversity in Ethiopia. Region such as, Somali, Harari, and Gambella were lower achievement of minimum dietary diversity. Eight supervised machine learning algorithms were trained and tested to accurately predict minimum dietary diversity among children. The random forest machine learning algorithm outperforms the other models used in this study in terms of predicting minimum dietary diversity. In this study, the top ten predictors demonstrated by the Beeswarm plot revealed that having children who were born at home, being female household heads, having a child between the ages of 12–17 years and 18–23 years, having children living in 7–10 family members, and living in rural areas increased the likelihood of meeting inadequate minimum dietary diversity. However, having an improved water source and having two under five children decreased the likelihood of meeting inadequate minimum dietary diversity among children aged 6–23 months. Despite the growing improvement in diet diversity among children in Ethiopia, with large inequalities across regions and socio-economic levels still prevailing. Efforts aimed at the marginal districts, increased access to clean water, and support for vulnerable households, notably female-headed households, are required. Predictive models can be used to spot vulnerable groups but must be applied considering subgroup performance so as not to provide an unequal intervention to all groups.

This study provides evidence on how to leverage machine learning techniques in large datasets, and provides insights for expanding and strengthening community-based nutrition initiatives aimed at educating caregivers about the need for nutritional diversity, and focuses on targeted nutritional interventions aimed at regions and demographics with lower dietary diversity. In addition, health extension workers should endeavor to improve antenatal care follow-up, promote facility delivery and family planning and provide nutritional counseling on proper feeding habits such as exclusive breast feeding, dietary diversity, and the use of appropriate meal frequencies for their children.

### Ethics approval and consent to participate

DHS data are available to the general public in various formats upon request from the Measure DHS website at http://www.measuredhs.com. We made a request to the measure DHS, briefly outlining the objectives of this analysis, and were granted permission to download the maternal datasets.

## Supporting information

S1 Fig Sensitivity analysis of imputation methods by using logistic regression model as a baseline for minimum dietary diversity among children aged 6–23 months in Ethiopia (EDHS 2005–2019, N = 8996). (DOCX)

S2 Fig Regional variation in predictor importance for inadequate minimum dietary diversity among children aged 6–23 months in Ethiopia (EDHS 2005–2019, N = 8,996). (DOCX)
