# Supplementary material for: Leveraging machine learning algorithm to predict minimum dietary diversity among children aged 6–23 months in Ethiopia
Source: PLOS Glob Public Health. 2026 Feb 26;6(2):e0005995. doi: 10.1371/journal.pgph.0005995 (PMC13030623; doi:10.1371/journal.pgph.0005995)
Supplement: S1 Fig — (DOCX) [file pgph.0005995.s001.docx]

S1 Fig. Sensitivity analysis of imputation methods by using logistic regression model as a baseline for minimum dietary diversity among children aged 6–23 months in Ethiopia (EDHS 2005–2019, N= 8996)
