# Supplementary material for: Leveraging machine learning algorithm to predict minimum dietary diversity among children aged 6–23 months in Ethiopia
Source: PLOS Glob Public Health. 2026 Feb 26;6(2):e0005995. doi: 10.1371/journal.pgph.0005995 (PMC13030623; doi:10.1371/journal.pgph.0005995)
Supplement: S2 Fig — (DOCX) [file pgph.0005995.s002.docx]

S2 Fig. Regional variation in predictor importance for inadequate minimum dietary diversity among children aged 6–23 months in Ethiopia (EDHS 2005–2019, N=8,996).


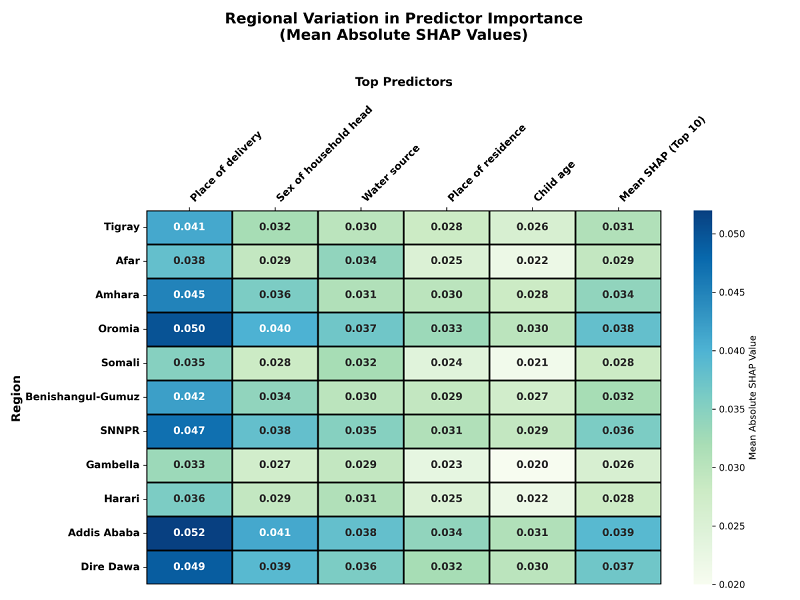


**Note:** Color intensity corresponds to average absolute SHAP values from the Random Forest model, with darker colors showing higher influence on prediction. Regional differences show the heterogeneity of influencing factors for dietary diversity across contexts.
